# A qualitative study of adolescent girls’ experiences of menarche and menstruation in rural Tamil Nadu, India

**DOI:** 10.1080/17482631.2020.1845924

**Published:** 2020-11-17

**Authors:** Anise Gold-Watts, Marte Hovdenak, Marguerite Daniel, Subramanian Gandhimathi, Rajamani Sudha, Sheri Bastien

**Affiliations:** a Department of Public Health Science, Norwegian University of Life Sciences, Ås, Norway; b Department of Health Promotion and Development, University of Bergen, Bergen, Norway; c Department of Community Health, Sri Narayani College & School of Nursing, Thirumalaikodi, India; d Department of Obstetrics and Gynaecology Nursing, Sri Narayani College & School of Nursing, Thirumalaikodi, India; e Department of Community Health Sciences, University of Calgary, Calgary, Canada

**Keywords:** Menstrual hygiene management, adolescent, gender norms, water, sanitation, hygiene, India

## Abstract

**Background**: In low- and middle-income countries, women and girls experience menstrual hygiene management-related health and social challenges such as urinary tract infections, social stigma, and school and workplace absenteeism.

**Purpose**: In this study, we sought to explore how adolescent girls in rural Thirumalaikodi, Tamil Nadu, India experience menarche and menstruation, how their experiences connect to the sociocultural context, and what strategies they use to manage menstruation. This study also informed the adaptation and development of a school-based water, sanitation, and hygiene intervention.

**Methods**: We conducted ten semi-structured qualitative interviews with adolescent girls in ninth standard from June-July 2018. Data were analysed using a thematic network approach.

**Results**: Findings revealed that menarche inaugurates biological transitions of puberty and cultural codes that shape gender norms. Gender norms in turn generate, maintain, and reproduce stigmatizing attitudes, beliefs, and practices that influenced the development of coping mechanisms at home and at school. Resulting adaptations to the intervention consisted of two activities (school lesson and an extracurricular activity) that address knowledge gaps and myths.

**Conclusions**: This study demonstrates the importance of qualitative research in unpacking adolescent girls’ experiences with menarche and menstruation. Study findings also show how formative research can contribute to the adaptation and development of a contextually and culturally-relevant water, sanitation, and hygiene intervention.

## Introduction

Adolescence marks a time of physical, emotional, cognitive, and behavioural growth and development. In particular, during the time of adolescence, girls experience the onset of menarche, signifying a transition from girl to woman (Dhingra et al., 2009). However, for girls in India, adolescence can often be especially challenging because for some, menstruation is linked to cultural beliefs, traditions, and stigma (Goel & Kundan, 2011). Whilst there has been increased focus on the importance of incorporating menstrual hygiene management (MHM) within health promotion interventions aiming to address water, sanitation, and hygiene (WASH) challenges, there is a need to develop a better understanding of what approaches are effective and in which contexts. This is an issue of substantial public health importance, given that adolescent girls especially, may face a variety of MHM-related health and social challenges such as urinary tract infections, reproductive tract infections, social stigma, shame, and school absenteeism (Das et al., 2015; McMahon et al., 2011; Priya et al., 2016; Ravi et al., 2016; P. Sharma et al., 2008) pertaining to insufficient puberty education, sanitation coverage, and access to menstrual absorbent materials (Hennegan et al., 2019; Sahin et al., 2015; Sommer et al., 2013). The development of effective health promotion interventions to address MHM, includes menstrual education and access to absorbents, soap, clean water, and private sanitation facilities (toilets and place for absorbent disposal) (Sommer & Sahin, 2013). These elements allow for the safe, supportive, and dignified management of menstruation to maintain health (Sommer, Caruso, et al., 2016; Sommer, Chandraratna, et al., 2016). Therefore, it is critical for WASH health promotion interventions to address the complex relationship between gender and sanitation, as well as the sociocultural context in order to develop interventions and strategies that improve MHM in low- and middle-income countries (LMICs) (Khanna & Das, 2016; Tilley et al., 2013).

In India, the topic of MHM is often linked to stigma, taboos, and restrictions, possibly stemming from the belief that menstrual blood is polluting (Garg et al., 2001; Omidvar & Begum, 2010; Puri, 2002). Studies have found that women and girls report not attending work or school because of menstrual-related cultural customs or taboos (Vashisht et al., 2018). This can have profound distal effects on their educational, economic, and social development (Caruso et al., 2017; Hennegan & Montgomery, 2016), demonstrating the necessity of WASH health promotion programming that prepares adolescent girls for the emotional, social, and physical changes in their body.

In 2010, India mandated that education was a fundamental right of every child with the instalment of the Right to Education Act, however a widespread lack of clean, private and safe sanitation facilities and education throughout the country continues to undermine this right for many adolescent girls (Sahin et al., 2015). In recent years, the Indian government has recognized the importance of proper MHM for educational attainment among adolescent girls and accordingly has developed initiatives to address MHM by improving sanitation in schools (Sahin et al., 2015). Such initiatives are included within the nation’s key sanitation strategy: Swachh Bharat Abhiyan (Clean India Mission) in the form of the National Guidelines on Menstrual Hygiene Management which provide “action” and “technical” guides to ensure schools have gender-segregated toilets to fulfil the need for privacy, and support adolescent girls during menstruation (S. Sharma et al., 2020). Systematic reviews have demonstrated that the availability of adequate sanitation facilities can improve school enrolment and retention among adolescent girls (Birdthistle et al., 2011; Jasper et al., 2012; Kuhlmann et al., 2017) however, adolescent girls still face several challenges regarding menstruation, including sociocultural beliefs and taboos that influence adolescent girls’ understanding of menstruation and management strategies (Dasgupta & Sarkar, 2008; Deo & Ghattargi, 2005; Nemade et al., 2009; Sahin et al., 2015; Selvi & Ramachandran, 2012). For example, existing research on beliefs about menstrual blood (as impure or polluting) affects how girls manage menstruation and encourage various restrictive practices (Dasgupta & Sarkar, 2008; Deo & Ghattargi, 2005; Nemade et al., 2009; Omidvar & Begum, 2010; Sahin et al., 2015). In a study from Tamil Nadu it is suggested that restrictive practices and taboos are perceived to maintain religious purity (Selvi & Ramachandran, 2012). Such restrictions and taboos include isolation and restrictions on participating in daily activities, using separate supplies such as a separate mat for sleeping, being banned from puja room (where ceremonies take place), using separate vessels, not touching flowers, or being in contact with men (Selvi & Ramachandran, 2012).

Despite expanding literature on MHM, there is still a lack of understanding of the complexities of this topic. MHM is often influenced by social and cultural beliefs that shape attitudes towards menstruation, which must be considered when designing WASH health promotion interventions and programmes. In addition, there is a need for an increased understanding of local menstrual beliefs and practices to help understand various constraints, restrictions, and pressures in a particular sociocultural context (Sommer, 2010). According to a systematic review and meta-synthesis of qualitative literature on women and girls’ experiences of menstruation in LMICs, there are also research gaps pertaining to the understanding of individual personal experiences, due to studies tending to rely on focus group discussions, which often divulge more collective experiences (Hennegan et al., 2019). Therefore, further research on adolescent girls’ personal experiences with menstruation may shed light on important sociocultural insights that can lead to more effective and sustainable health promotion intervention planning and adaptation (Phillips-Howard et al., 2016).

This study was informed by preliminary findings of other unpublished formative research activities (e.g., a curriculum assessment and semi-structured key-informant interviews with local stakeholders), which are not included in this manuscript that suggest that MHM is an important WASH-related concern among stakeholders from local healthcare and educational institutions. Therefore, this formative study engaged adolescent female students to understand experiences of menarche and menstruation, in order to adapt a school-based WASH health promotion intervention: Project SHINE (Sanitation and Hygiene INnovation in Education) (Bastien et al., 2016).

### Research objectives

The objectives of this formative research were to explore 1) how adolescent girls in the rural community of Thirumalaikodi, Tamil Nadu, India experience menarche and menstruation; 2) how their experiences connect to the sociocultural context; and 3) what strategies they use to manage menstruation. These contextual and cultural insights are essential in order to adapt Project SHINE to the southern Indian context.

### Theoretical grounding

The use of a theoretical lens helped shape the research questions, data collection, and analysis (Creswell, 2014). The study was informed by a feminist perspective, to shed light on gender-specific sanitation challenges and needs. The incorporation of the feminist perspective also allowed for exploration of gender-related experiences and the lived experiences of women. Feminist research aims to understand and challenge how individuals experience the world and through this understanding help facilitate change for marginalized or vulnerable groups, such as adolescent girls in India (Creswell, 2014; Reinharz & Davidman, 1992). The feminist perspective was used in this study to explore the following conceptual ideas: 1) menarche and menstruation are a biological event that only women (girls) can experience and 2) recognition of the societal constructions of gender. Thus, in this study a feminist perspective is used to highlight the importance of the sociocultural context and its influence on adolescent girls’ experiences of menarche and menstruation.

Gender must be understood within the social structure in which it was constructed, negotiated, maintained, and reproduced (Heise et al., 2019). These social structures are supported by gender norms which are “rules that govern the attributes and behaviours that are valued and considered acceptable by men, women, and other gender minorities” (Heise et al., 2019). Butler (2003), argues that gender is not essential or natural, but rather forged from continuous gendered performances. Understanding gender as performative provided an entry point into understanding the complexities of gendered experiences. Moreover, the application of a feminist perspective informed our understanding of adolescent girls’ experiences of menarche and menstruation, how gender is constructed, negotiated, maintained, and reproduced, and how these experiences connect to, and are shaped by the sociocultural context.

## Materials and methods

### Setting and sample

The study presented in this paper, was part of the formative research phase in the process of adapting Project SHINE and took place in the village of Thirumalaikodi which is located in the Vellore district of the southern Indian state of Tamil Nadu. Two schools from Thirumalaikodi village were selected to participate in the study based on expressed interest in Project SHINE demonstrated by the local community. A sample of 10 adolescent girls in ninth standard (grade nine), who averaged 14 years from two schools in the rural community of Thirumalaikodi were invited to participate in semi-structured qualitative interviews from June-July 2018. Adolescent girls were the selected target group for this study because of their unique position to reflect upon their experiences with menarche and menstruation. Furthermore, adolescent girls in India are especially vulnerable to a range of adverse health and social outcomes such as urinary tract infections, reproductive tract infections, social stigma, shame, and school absenteeism (Dasgupta & Sarkar, 2008; P. Sharma et al., 2008).

In this study, adolescents who attend school in Thirumalaikodi, primarily reside throughout the Vellore district, and commute to school six days a week either by foot, autorickshaw, motorbike, or school bus. Both schools selected for this study were English-medium private institutions however, a majority of the schoolchildren enrolled at these schools are the first in their family to read and write. Furthermore, and most notable, the temple and local spiritual leadership which draws on Hinduism played a large role in education at the schools, thus reflecting local spiritual beliefs and values. For example, yoga, meditation, Vedic chanting, and traditional dance are regularly practiced at school. Additionally, at the time of research, there was not an active curriculum focused on MHM at either school.

Although there is little information published about Thirumalaikodi, information regarding the Vellore district itself (including both rural and urban areas), is more broadly available. In the district of Vellore, there are approximately 3,9 million people who reside in 22 Town Panchayats and 858 villages (Directorate of Census Operations Tamil Nadu, 2011). According to the Indian census, in Vellore, 22% of the population identified as Scheduled Caste, while 2% identified as Scheduled Tribe, there is a literacy rate of 79%, and 57% of the population reported that they are not engaged in any type of employment (Directorate of Census Operations Tamil Nadu, 2011).

A purposive sampling strategy was employed to recruit participants for the study, which focused on the inclusion of girls who had already begun menstruating and included similar characteristics as future SHINE intervention participants (e.g., enrolled at the same school, similar age, and locality). Verbal and written information about the purpose of the study was provided to girls before participating. First, adolescent girls were informed of the study through the research project’s local community liaisons (two schoolteachers) at each school. Community liaisons informed students about the study by explaining that, researchers from Project SHINE were interested in speaking to them about their experiences with menstruation and their participation was entirely optional. If they indicated interest, they were invited to participate and asked to obtain written consent from their parent or guardian. Selection criteria for participation included the following: 1) must currently attend school in ninth standard, 2) be comfortable expressing themselves in English, and 3) attained menarche (onset of menstruation). Given that interviews were conducted in English (the primary language of instruction at the school) and not other local languages (e.g., Tamil or Telugu), adolescent girls were informed that interviews would be conducted in English prior to agreeing to participate. Although students were purposively sampled to mirror the future SHINE intervention’s potential participants at the school, due to the nature of the study, representativeness was not prioritized. Instead, participants were purposively sampled to meet selection criteria in order to generate rich, in-depth qualitative data of participant’s lived experiences.

A strong, researcher-participant relationship that established trust and rapport was key to sampling and data collection in this study. There was no existing relationship between researchers and participants at the time of data collection, however, the research team worked to cultivate trust and respect through other informal exchanges. For example, AGW and MH were introduced to students by the school principal at an all school assembly and frequently visited the school prior to data collection. Furthermore, the research team utilized assent forms and brochures to provide a clear understanding of the research project’s main aims to nurture trust between researchers and participants. For example, assent forms and a study brochure were given to students which explained the specific aim of this study and provided additional information on confidentiality.

### Ethical considerations

Ethical approval was obtained by the Norwegian Centre for Data Research (reference numbers: 53162/54618) in Norway and the Institutional Ethics Committee/Institutional Review Board at the Sri Narayani Hospital and Research Centre (reference number: 30/25/02/17) in India. Due to the age of participants and the nature of the discussions, the research team set up several measures to ensure participants and parents fully understood the study requirements before agreeing to participate. First, assent from minors and written parental/caregiver consent was obtained. Then interviews were conducted in secured rooms at the school to ensure the privacy of participants (only participants and researchers were present during interviews). In order to protect participants’ confidentiality, they were also reminded of their right to withdraw from the study at any time, the right to decline to answer questions, as well as their right to choose to turn the audio recorder off at any time throughout the interview.

### Data collection

A qualitative, phenomenological approach with narrative interviews was employed (Elliott, 2005), so that questions were developed to help prompt participants to tell their personal stories. Semi-structured qualitative interviews were conducted by the first and second authors (AGW and MH), both female researchers with experience in qualitative research methods who had previously spent several months in the community. Since the interviews dealt with a potentially sensitive topic, the research team received cultural guidance from local experts on MHM prior to data collection (e.g., appropriate framing and terminology). Experts also recommended that we refrain from asking students explicitly about which caste they belong to because it is a taboo topic that may cause distress and requires comprehensive follow-up. Interviews lasted for approximately 30–45 minutes and were guided by a five-question interview-guide. The interview guide was developed based on previous research conducted by MH in the community (Hovdenak, 2018). Interviews first explored constructions of gender, then participants were asked to share stories of their first period, followed by discussions about various experiences relating to menstruation. After the completion of ten interviews and three follow-ups, we determined that little new information was being generated therefore we had reached thematic data saturation.

Semi-structured interviews were audio recorded and transcribed verbatim; however, participants had the option to turn off the audio recorder at any time, and three declined to be audio recorded (7 out of 10 participants consented to have their interviews audio recorded). During transcription, identifying information was removed to maintain the confidentiality of the participants (e.g., names, dates, and places). For all interviews that were not audio recorded, the interviewer took detailed notes to capture experiences.

### Data analysis

Data were analysed using Attride-Stirling (2001) thematic analysis approach in conjunction with Dedoose software for managing qualitative data. First, we (AGW and MH) coded the data inductively. Then, codes (basic themes) were mapped and condensed into organizing and global themes, which will be discussed in further detail (Attride-Stirling, 2001). In addition, in order to increase rigour, we engaged in an inter-coder reliability exercise in which two members of the research team (AGW and MH) coded the same transcript, compared codes, and discussed their application of the codes, to ensure that there was a common understanding of codes, how they were defined, and applied. Analysis was also informed by a feminist perspective (Butler, 2003; Olesen, 2011; West & Zimmerman, 1987). The outcome of the analysis is shown in Table I.

**Table I.** Thematic map outlining the development/evolution of codes to global themes

Global Themes	Organizing Themes	Basic Themes (Codes)
Emotional duality of menstruation	Positive experiences	Rite of passage celebration
		Knowledge and understanding
	Distressing experiences	Taboos
		Psychological distress in different contexts
Negotiating “womanhood”: transitions and challenges	Gender norms and restrictions	Learning from mothers and grandmothers
		Impact on religious practices
	Accepting adult responsibilities	Loss of childhood identity
		Relationships in transition
Social navigation strategies while menstruating	Concealment and secrecy	Maintaining outward appearances
		Do not tell
	Self-imposed isolation	Non-participation at school
		Avoiding public spaces

## Results

Table I outlines the thematic map that comprises this study’s findings (as presented below). The illustrative excerpts displayed are provided to highlight themes; however, they have been edited for readability. Edits included the removal of identifying information, paraphrasing, and enhancement of grammatical correctness since interviews were conducted in the participant’s second language. For example, “I not listen” was changed to “I [do] not listen”. Changes are indicated by square brackets.

### Menstrual paradox: the emotional duality of menstruation

Girls reported both positive and negative experiences of menarche and menstruation, this theme describes the conflicting emotions participants discussed. Often, positive experiences were associated with menarcheal rites, or a celebration described locally as a “function” in which girls are honoured by their family and friends over the course of several days. While this ritual may serve several purposes (e.g., relaying that a girl is now ready for marriage), the narratives presented by the participants focused on the celebratory nature of the experience. At menarche, girls also reflected retrospectively upon concerns, fears, and distress felt regarding the biological and social changes they endured. Therefore, we developed two organizing sub-themes which included “positive experiences” and “distressing experiences”.

#### Positive experiences

Adolescent girls described the *function* as a “happy” and “enjoyable” experience. They spoke about several practical components of the function, such as rituals and traditions performed, however, it was emphasized that missing school, receiving gifts (e.g., new dresses, saris, and gold), and seeing their extended family were their favourite aspects. One participant further elaborates:

When we have periods, they will conduct the function, [with] our family [and] relations. They kept one basket on my head and then they [poured] water [on] me and a good moment happened, and I was so happy. A girl, I think, sometimes will be happy, sometimes I feel bad, but it is also a positive thing (Participant 10).

This excerpt illustrates how the function was a celebratory event for the girls as they express their elation with the activities and traditions that transpired.

Although many positive experiences centred on the function, one participant described the time of menarche in more general terms, as a natural event and opportunity for informing and promoting understanding:

… Girls should not be scared of this, it’s just a part of their life. If they get depressed on this, how will they face all the [other] situations … Because every girl will be getting this, [wherever] she is from, whatever the caste or whatever the country. She will be getting it. So, she should not think like, ‘what will they think [about] it?’ If it is spotting, she should be conscious and use the napkins. It should be in a proper size and everything. Nowadays everything is just developing, even the napkins are the correct size of the panty, so [there is] nothing to worry about. So, girls should not feel afraid for coming to school or wherever they’re going (Participant 5).

Here the participant described menstruation as a naturally occurring phenomenon among all girls. She normalizes menstruation (confronting negative discourses of fear and shame), by highlighting that experiences of menstruation transcend socially stratifying structures such as nationality and caste. She also discusses the proper use of menstrual absorbents to avoid spotting and to feel comfortable in school, further reflecting upon how innovations are currently being developed to meet needs of girls, thus positively influencing MHM.

#### Distressing experiences

Girls also shared how menstruation adversely impacted their everyday lives through attitudes and beliefs that perpetuate taboos that incite concerns, distress, or fear. Often, participants entwined menstruation with distress. Distress may also reference psychological indicators such as sadness and stress. For example, girls were reportedly told that improper disposal of menstrual absorbents would attract wild animals, thus revealing when they were menstruating to others (a distressful thought). They were also told that ghosts haunt women during menstruation. One participant describes how she manages the ghosts by sleeping outside during menstruation, while others discussed the importance of carrying a copper nail to thwart them away. Such taboos may lead to restraint and feelings of distress, or concern. This was illustrated in the following:

We should not water plants … if we maintain a distance between the plant it is OK. [However] we should not touch, because, the plant will suddenly shrink, because it can’t tolerate the heat [from] us. Women during that particular three to five days (during their period), we have more heat. It is prove[n] in science (Participant 5).

This participant describes how cultural beliefs regarding the heat tolerance of plants foster concerns that her menstruating body can potentially cause harm; therefore, she refrains from touching plants during the first days of her menstrual cycle.

### Negotiating “womanhood”: transitions and challenges

Often girls discussed how they were taught to follow various “rules” or cultural codes as newly menstruating women. These norms restrict behaviour, social relationships, and personal conduct, which lead to the formulation of the global theme, “negotiating ‘womanhood’: transitions and challenges” with an organizing themes, “gender norms and restrictions” and “accepting adult responsibilities”.

#### Gender norms and restrictions

Throughout interviews, study participants described several “rules” or codes which entailed specific ways of dressing, acting, and interacting which we classified under the organizing theme, “gender norms and restrictions”. Girls frequently reported engaging in gendered performances, which encompass acting “feminine”, abiding by “rules” or cultural codes, and engaging in appropriate conduct in their new identities as women. One participant shared that “rules” were often introduced at menarche when she shared that, “many rules was there when the first periods occurs (Participant 7)”. For example, during their period girls described practicing bathing rituals (e.g., head bath^1^), being restricted from spaces such as the *puja*^2^ room at home and temples, refraining from cooking food for others, being segregated from other members of the household, and being instructed to use a designated plate for eating. One participant shared that during this time, she “will pray in my home itself, but not enter in the puja room (Participant 6)”.

Several participants described not knowing about menstruation until they attained menarche. For example, one participant shared that she did not know she even had her period until after two days when her mother did her laundry, demonstrating a lack in basic menstrual-related knowledge. Although several participants did not expect the biological changes they would endure, “rules” or cultural codes were prioritized post-menarche. Girls reported that family members, especially their mothers or grandmothers, often taught them “rules” or cultural codes when they attained menarche, which governed attitudes, beliefs, and practices. One participant shares how these “rules” or cultural codes shape gender norms when she explains:

She gave information about how to be proper, how to sit, and how to behave. We have to be studious, we have to remain a gap, and if we share something, you must think twice. You can’t share everything to everyone (Participant 10).

Participants mentioned several examples of how gendered norms impacted religious practices. Norms often influenced the manner in which girls adapted as newly menstruating women. The following excerpt describes how restrictive gender norms may have further implications when one participant shares:

On my third time, when I got [it] I was not [aware of] the 28 days gap. We were out, and suddenly [while] traveling I got [my period]. I was not prepared for it. So, my trip was fully disturbed. Because of this, I should not go to temples. We had a plan of going to four temples the next day. [But], ‘til the fourth day, until the period is controlled, we should not go to temple or worship God. [So] the trip cancelled, [and] because of me, no one went. Everyone was with me, so it disturbed me. Firstly, when I hear the news that we’re not going to go anywhere, we have to stay at home, I was a little bit stressed. Like, what is this? [I think] because of me everyone is going to get moody. So, I thought if I would have stayed in my home, everyone will be enjoying. And second, I was disturbed because I could not go and experience all the places, like what we planned (Participant 5).

This quote illustrates how externally enforced restrictions of not being able to attend the temple can also be distressful when she describes her desire to attend the family trip, but the entire family was forced to turn around because she was not allowed to enter temples. Furthermore, this participant emphasizes the guilt she felt when she blames herself for the trip’s cancellation and wishes that she had stayed home so the rest of the family could go.

#### Accepting adult responsibilities

As girls enter womanhood, they describe shedding their childhood identity. In many ways, childhood is connected to freedom, and post-menarche “rules” establish codes of conduct to be followed that promote a separation between genders or gendered behaviours. Several participants discussed how gender roles were enforced after menarche, which promotes shifts in their identity. One participant describes the transition as an advancement into adulthood when she says,

When I was small, my mother [would] clean all things. I [was] enjoying the small age. Then when I’m coming on ninth standard, and I get periods, oh, why, it’s very difficult. I feel difficulties, after that periods. Before periods, I was really enjoying that age and I was playing with everybody, boys … girls. And, before periods I will wear normal [clothing], now I have to cover everything (Participant 6).

Other participants echoed similar sentiments by elaborating on how the adoption of cultural codes enforces gender norms that restrict behaviours in womanhood:

It completely changed. When I was before the age (of menarche), I had all rights to go here [and] there and there [were] no restrictions. After the age of adolescences, restrictions were, not about talking to someone, but it was physically. Like, you can’t go everywhere you want. You will need a guide to guide you, ‘til you complete your teenage [years]. So, a little bit of restrictions [were] raised. And I also had responsibility in my home. And contact to males, like gents, we have to have a gap (Participant 5).

Both excerpts illustrate the transition of identity after menarche. The participant describes changes as a swift descent from freedom to the staunch adherence to various cultural codes. She expresses her difficulty in dealing with this transition and loss of freedom.

Participants revealed that gender norms had a profound influence on their conduct, which affected relationships with others. Moreover, participants suggested that the relationships most affected were their relationship with men in their lives such as neighbours, friends, cousins, and their father. One participant shares these relationship changes after menarche:

Yes, my life has changed totally when [the] first period occurs, and my mom, and my sister, my grandmother, all said you should not play with boys, you should not go outside regularly, you should not play with all, but you should not talk to all. You should be in limit, and the fathers also be in their limit. Before they are very friendly with us, but [when] the period occurs they will not [be] friendly with us [any]more. I felt very sad of that. I felt very sad. My brother, my father, all are separated, [there is a] little bit distance [between] us and them (Participant 7).

Here, this participant talks about the vast transition her life underwent after menarche, most notably the enforcement of gender-influenced attitudes, beliefs, and practices (e.g., separation between genders). The participant expresses dismay and sadness that she will no longer be able to maintain relationships with important people in her life because of their gender (e.g., brother and father). The same participant continues:

[Before], we can talk, we can enjoy, we can go anywhere, we can play, we can do anything with our father. But [when] periods occurs, all things will stop, and [a] limit is there. [A] little bit [of] distance also will be there. When we are sitting, we can feel the distance of our father and it feels so bad too. Mainly the olden [generations] they are following these rules and taking to our modern world. It is not good, I think so. We can’t separate and limit, it’s not nice, I feel very bad (Participant 7).

### Social navigation strategies while menstruating

The global theme, “strategies to navigate menstruating body” describes various coping mechanisms that participants developed to cope and manage menstruation. We identified two organizing themes, which include “concealment and secrecy” and “self-imposed isolation”.

#### Concealment and secrecy

Participants frequently shared how their periods were a private matter, not to be shared with others. One participant explains, “Whether we have periods or not, we should not tell others. It’s embarrassing (Participant 1).” Participants also developed personal approaches to conceal menstruation, which included secrecy and isolation. One participant describes how she copes with menstruation by concealing it from peers and family:

But my friends cannot identify whether I have periods or not. One basic thing ma’am, girls are keeping black *bindi*, [when] they have periods. … And in our family, also, girls we should keep only black [during] our period days. We should not keep that sandal, kum kum,^3^ any of that. Only black *bindi*. I don’t like. My friends are teasing. So, whether [I have] periods or not, I don’t tell (Participant 1).

Here, the participant describes the routine practice of women wearing a black *bindi*^4^ during menstruation. However, when she is menstruating, she avoids wearing this *bindi* to conceal or hide her menstrual status from others, including her grandmother. She also discusses the embarrassment she feels when she gets her period mainly because of teasing and taunting from peers, alluding to the implications of cultural practices on her psychosocial wellbeing. Therefore, she has developed a strategy of secrecy that helps her cope with the shame and stigma she experiences during menstruation. By choosing not to wear her black *bindi* she resists this cultural practice. However, it is unclear if this resistance is rooted in denial of the auspiciousness of the practice, or if she has prioritized protecting herself and concealing her menstruating body. Either way, this excerpt illustrates an example of an approach to concealing menstruation and how participants may feel uncomfortable sharing their menstruation with others.

#### Self-imposed isolation

Participants also discussed internally-imposed withdrawal and isolation as an approach to cope with menstruation. Withdrawal can lead to feelings of loneliness, failure to develop healthy attachments to others, and failure to participate in various community, academic and work activities. Throughout interviews, participants described self-inflicted withdrawal from school, social environments, and relationships. One participant reveals:

[At] school we don’t discuss this. When someone is so sad, in this time, in our school, my good friends, they’ll just sit alone, they’ll not mingle into the group. They just sit alone, and they just feel sad. I personally will ask what happened, they’ll say, “I got periods”. Why are they not coming out? They are afraid if they stand, something will happen on their dress. So, they are personally disturbed. In this way, they are disturbed mentally, physically. They can’t concentrate even on studies (Participant 5).

Here, the participant describes a lack of openness around the topic of menstruation among her peers. Girls at school will withdraw from activities (due to fear of spotting on their clothes). This withdrawal and isolation during menstruation impacts both academic achievement and psychosocial wellbeing. For example, one participant describes an experience with her period at school:

[During my] first period, my teachers [asked] me, “Why are you late?” [However] I’m not seeing [my teacher], I’m thinking about, why [is] this problem coming now! I’m thinking about that only. For that class, I [do] not listen, [I am] confused. When I go home, and [read], I’m confused. I can’t learn the lesson. In that time, I am not listening [in] class. That time I say [to myself] why is God doing like this? I am thinking about that only. That time I’m [scared], very [scared] (Participant 2).

The participant discusses having difficulty focusing in school when she has her first period, which affected her school performance and ability to fulfil class assignments. Here, her inability to cope with menstruation leads to distress and fear.

Participants also discussed how restrictive gender norms also influenced internally-imposed withdrawal. One participant describes:

When I get periods, I avoid going to shops. Because many of the male guys will [be] in that place and then I don’t know how to be still a girl. I want to become a brave woman so, that I can be maintaining my distance with all of those [men] who are aged. When I am in [my] home, I feel so lonely, because always I am home. I do not like to [be] with men, like aged. I think it’s dangerous for me. Something will happen. So that I did not go (Participant 10).

This excerpt describes the internally-imposed protective measures or strategies she has developed to avoid contact with adult men. Moreover, she suggests that brave women are able to properly maintain a distance with men, describing contextual cultural codes, thus demonstrating how her construction of bravery or fearlessness is still significantly informed by gender norms.

Another participant shares how internally-imposed protective measures potentially impact academic achievement and school attendance:

I used to say [to] my friends, like, “This is just a part of life, so we should not get depressed on this particular thing.” But other girls are really afraid of coming to school, whether someone will see us, or something will look odd … (Participant 5).

Here, this participant illuminates how motivation to conceal menstruation from others affects other aspects of everyday life.

## Discussion

This study revealed that adolescent girls’ experiences of menarche and menstruation are largely shaped by cultural codes and gender norms, which further influence gendered attitudes, beliefs, and practices that construct and maintain conceptions of womanhood that alienate, isolate, and restrict.

Among participants in this study, menarche attainment was perceived as both positive and distressing. Positive aspects reported focused on the traditional practices associated with menarche (menarcheal rites). Girls described how they felt happy or joyful because of celebratory menarcheal rites of passage into womanhood, during which, family members gave the girls gifts of clothing and gold. An emphasis on the positive aspects of menarche is at odds with other studies that often characterize menstrual-related experiences as negative, thus demonstrating the complexities and paradoxes within adolescent experiences of menarche and menstruation. This finding is also consistent with other literature that documents menarche as a celebrated rite of passage (Buckley & Gottlieb, 1988). However, these rites still exist within an overall discursive positioning of menstruation, which highlights distressing experiences. Researchers have previously explored experiences of women and girls with menarche and menstruation through a feminist lens, and how these experiences may shape their identities and role as women (Burrows & Johnson, 2005; Roberts et al., 2002; Sommer et al., 2015; Tilley et al., 2013). Furthermore, feminist research highlights how negative experiences with menarche and menstruation may be influenced by the sociocultural and historical context which heavily influences cultural attitudes and beliefs (Burrows & Johnson, 2005).

Girls also reported several cultural codes that influenced their personal experiences. These cultural codes encompassed cultural and spiritual rituals and practices such as bathing rituals or a “head bath”, which would alleviate physical symptoms associated with menstruation and ensure purification, hygiene, and cleanliness. In addition, several participants described how they did not attend temple or enter the puja room at home during menstruation, revealing that these beliefs inform practices in their everyday lives. It was also explained locally, that girls were prohibited from attending temple, since they are believed to be incapable of withstanding divine energy. Furthermore, participants discussed avoiding contact with plants, as well as other practices such as sleeping outside to manage ghosts, refraining from cooking for the household, or using a designated plate for eating, which demonstrates how culturally-informed attitudes, beliefs, and practices influence adolescent girl’s personal experiences with menstruation. Other research from Tamil Nadu and different contexts and cultures has suggested that cultural codes post-menarche also promote similar gender norms and rituals. For example, findings from a multi-site study reveal that respondents reported they believed menstruating women should not visit temples or religious sites because of their impure state (Snowden et al., 1983). Another study from Tamil Nadu, also suggests that restrictive rules influence dietary habits such as avoiding non-vegetarian foods (e.g., meat, fish, and eggs) at menarche (Ferro-Luzzi, 1980).

Girls developed mechanisms to manage menstruation such as internally-imposed strategies to cope with or conceal menstruation, which often included secrecy, isolation, and withdrawal. Indications that menstruation should be kept secret, suggests that menstruation is perceived as an embarrassing or shameful occurrence by some study participants. Throughout interviews, girls discussed their efforts to conceal menstrual bleeding. For example, one participant shared how she refused to wear a black *bindi* to suppress any visual cues, which may disclose that she is menstruating to others. *Bindis* are an auspicious mark or dot that is traditionally worn by Hindu women, however in this context, we observed women of different faiths performed this ritual practice daily, to protect the Sixth Chakra or Third Eye. Thus, demonstrating the cultural and spiritual significance of *bindis* in this context. Other studies in other Indian contexts, also described MHM practices that are motivated by a desire to conceal or hide menstrual blood. However, as illustrated by this example, girls emphasize the importance of concealing strategies in order to prevent others from knowing they are menstruating. Another study in Mumbai, also discusses concealment. This study revealed that girls dried menstrual cloths under their clothes in order to conceal menstruation from male family members, while another study in Odisha reports that women use discretional strategies to eliminate lingering traces of menstrual blood when washing their clothes (MacRae et al., 2019; Muralidharan, 2019). Other feminist scholars discuss how experiences with menarche and menstruation among adolescent girls are frequently characterized by secrecy and concealment, and fuelled by cultural beliefs that suggest menstrual blood is impure (Britton, 1996; Burrows & Johnson, 2005; Lovering, 1995; Moore, 1995).

These cultural codes also had profound influences on familial relationships between genders (e.g., father/daughter sister/brother). At menarche attainment, girls were separated from the household in the lead up to their function (menarcheal rite), which foreshadowed changes in participants’ relationship with men in their families. Girls also discussed how they felt post-menarche, when cultural codes restricted their relationships with men, which led to social withdrawal and isolation. Here, study findings reveal how social constructions of gender shaped menstrual-related cultural codes and/or “menstrual etiquette” (Laws, 1991) that lead to gendered performances that maintain idealized distinctions between masculine and feminine (Ussher, 2006).

Participants also discussed how post-menarche, they were instructed (often by family members) to modify conduct, relationships, and clothing to successfully transition into womanhood. As one participant stated, it was explicitly communicated that as a woman, it was now important to act “proper” to maintain respect. Respect is important in this context and often entails respectful ways of greeting others, dress, gestures, and ritual. This is reflected in other research about menstruation. Garg et al. (2001) discuss how adolescent girls are told they must avoid behaviours, restrict interactions with men, dress modestly, and not touch religious texts or visit holy places. This further illustrates how menarche launches a transition into womanhood, which considerably impacts the lives of adolescent girls.

Throughout interviews, girls also discussed their experiences with menstruation in the context of the school. At school, various restrictions linked to menstruation may be discursively positioned to generate negative attitudes around what it means to be a girl, demonstrating how gender norms are affected during adolescence. Furthermore, participants discussed how they were uncomfortable talking to friends about menstruation because they feared menstrual-related bullying. In addition to teasing and gossip associated with menstruation, girls discussed how menstruation affected their social relationships and their ability to engage in school. This is consistent with other research on menstrual-related bullying, which demonstrates how fear of teasing is interlinked to menstrual odour or leakage (Mason et al., 2013). Moreover, other research reports that a lack of understanding from male classmates fosters an unsupportive social environment at school (Mahon et al., 2015).

Feminist research which explores experiences with menarche and menstruation focuses on how such experiences are constructed, negotiated, maintained, and reproduced through various social interactions that evolve from sociocultural representations and practices (Burrows & Johnson, 2005). Excerpts presented in this study demonstrate this link between experiences with menarche and menstruation and the local sociocultural context. Several participants described knowing little or nothing about menstruation pre-menarche. One participant shared how she did not know she was having her period until after the second day when her mother discovered bloodstains in her underwear. Several participants described information about menstruation being transferred inter-generationally through their mother, grandmother, or other family members. Other research has yielded similar findings, which suggest that insufficient knowledge about puberty, reproductive health, and menstruation is transferred inter-generationally, leaving adolescent girls poorly informed and ill-equipped to cope with menstruation (Chandra-Mouli & Patel, 2017; Mathiyalagen et al., 2017; Narayan et al., 2001).

Insufficient knowledge also may contribute to perpetuating negative interpretations of womanhood, leading to isolation, shame, and fear (Dasgupta & Sarkar, 2008). Based upon the type of knowledge emphasized and transferred by family members, we discern that this information contributes to the discursive positioning of menstruation as something negative. Drawing from the work of feminist scholar Lovering (1995), instead of labelling girls as “ignorant”, and faulting the mother or teachers for menstrual knowledge gaps, in this study, we also tried to reflect upon and consider how other factors may profoundly influence knowledge production and transfer. For example, adolescent girls may lack knowledge about menstruation because the sociocultural context positions menstruation as problematic, thus difficult to discuss with others. Furthermore, knowledge transfer may be minimal because cultural and historical beliefs make it embarrassing or taboo to discuss menarche, menstruation, and/or menstrual blood. Commonly, adolescents learn about menstruation from others, however it was revealed that menstrual hygiene education at school was limited. For example, some programmes that addressed MHM at the schools where research was conducted were no longer running at the time of data collection.

There are several strengths and limitations of this study. Prolonged engagement is a technique that helps establish rapport, strong relationships, and credibility among participants (Connelly, 2016). First, researchers attempted to establish rapport by spending time volunteering at the school, and by attending local festivals and events; however, due to the sensitive nature of this study, some participants still felt uncomfortable sharing their experiences and declined permission to have interviews audio recorded. However, efforts were made to ensure participants felt safe by explaining confidentiality procedures and securing a private room to conduct interviews. It is important to acknowledge some of the researchers’ status as outsiders to the community and the power imbalance that entails, as well as the effect this could have had on data collection. Therefore, it was crucial that we incorporated local capacity and expertise into the research design and manuscript writing processes to establish a cooperation that was culturally respectful, collaborative, and mutually beneficial (Tervalon & Murray-Garcia, 1998). The added value of the research team’s personal experiences from the field also informed and helped develop findings, however, we recommend a more rigorous integration of informant feedback (e.g., member checks) to help build a more rich, nuanced, and complete understanding of the context. Strengths also included the application of an intercoder reliability exercise to enhance consistency in coding among researchers, which also strengthened rigour of findings.

However, there were also some limitations with the study. Interviews were conducted in English, which, while the primary language of instruction at the school, is not the first language of the participants, and could have affected some participant’s ability to fully express their personal experiences. Language could have also influenced the clarity and flow of communication and analysis. It is also vital to acknowledge that the methods employed, and the sample selected may limit the ability to generalize findings to other contexts and populations. Nevertheless, this was not the purpose of this formative work, rather we tried to recruit a sample that would mirror future intervention participants. We hope this study provides rich contextual insights into this population, in addition to exemplifying how formative research can be used in the cultural adaptation process for school-based WASH health promotion interventions. However, we recognize the value of further exploration of adolescent girl’s experiences of menstruation and menarche with the inclusion of a more diverse sample (e.g., inclusion of participants from a diverse locality). Furthermore, this study utilized a feminist perspective, identifying it as an important lens for research regarding MHM. However, since male and female adolescents have very different experiences regarding menstruation, data from adolescent boys were not collected, thus limiting insights on adolescent boy’s menstrual knowledge gaps and commonly held beliefs. Therefore, we recommend further qualitative research (e.g., interviews, focus group discussions, or use of other participatory methods) to include those perspectives.

Findings from this formative work were used to inform the adaptation and development of school-based WASH health promotion intervention activities. This adaptation process utilized a combination of adaptation frameworks such as Mckleroy and Wingood’s Map of Adaptation Process (McKleroy et al., 2006) and Rescinow’s Cultural Sensitivity Framework (Resnicow et al., 1999). Based on findings, we adapted Project SHINE to include additional intervention components that address insufficient knowledge of menstruation, social support networks, and various attitudes and beliefs that perpetuate stigma and taboos at school.

In making adaptations to the original SHINE curriculum, we sought to specifically address information gaps and popular myths regarding MHM in this context. First, it was critical that intervention content be adjusted and include additional participatory activities focused on MHM, to coordinate with Project SHINE’s participatory nature (Bastien et al., 2016). For example, a peer-to-peer MHM activity was introduced which employed age-appropriate visual aids such as the Menstrupedia comics to address information gaps and other interactive teaching strategies (e.g., role play) to enhance social support networks that promote a supportive school environment. Additionally, another educational lesson with a special focus on MHM was included which utilized interactive games that aim to debunk specific menstrual-related myths and taboos that are prevalent in this sociocultural context (e.g., girls have the potential to contaminate/harm plants during menstruation). Tailoring and changes also addressed prevalent beliefs and practices that may be harmful to adolescent girls.

## Conclusion

This study highlights menarche and menstruation experiences of adolescent girls in rural India with a specific focus on generating an in-depth understanding of the local sociocultural context. Findings suggest that menarche inaugurates biological transitions of puberty and cultural codes or restrictions that cultivate rigid gender roles and norms in this context. Furthermore, gender norms also generate, maintain, and reproduce stigmatizing attitudes, beliefs, and practices that influenced the development of coping mechanisms related to menstrual hygiene management at home and at school. In addition, this study demonstrates the importance of qualitative research in unpacking adolescent girls’ personal experiences, which contributes to rich formative research for the adaptation and tailoring of WASH health promotion interventions to suit the local context.

## Data Availability

The datasets generated for this study are available on request to the corresponding author.

## Notes

1. Traditional bathing of the head and hair done during menstruation, practiced both for hygiene and to alleviate symptoms associated with menstruation.

2. Ritual acts of devotion and worship.

3. *Kumkum* is a red powder composed of lime and tumeric that is commonly applied to the centre of one’s forehead.

4. A *bindi* is a marking applied to the centre of the forehead, traditionally placed on the sixth chakra, or third eye.
